# Tumor Necrosis Factor-Alpha Gene Promoter Region Polymorphism and the Risk of Coronary Heart Disease

**DOI:** 10.1155/2013/203492

**Published:** 2013-12-05

**Authors:** Gul Zareen Asifa, Afrose Liaquat, Iram Murtaza, Syed Ali Raza Kazmi, Qamar Javed

**Affiliations:** ^1^Department of Biochemistry, Quaid-i-Azam University, Islamabad 45320, Pakistan; ^2^Institute of Biomedical and Genetic Engineering, Islamabad 2891, Pakistan

## Abstract

*Background*. Tumor necrosis factor-alpha (TNF-**α**) gene polymorphisms have been implicated in the manifestation of atherosclerosis. Controversy exists regarding the link between the cytokine's variant genotype and CHD among different ethnic groups. There have been fewer studies on the TNF-**α** gene −1031T>C and −863C>A polymorphisms in relation to CHD. Therefore, the current study was designed to investigate the association of the TNF-**α** gene −1031T>C and −863C>A polymorphisms with CHD in a Pakistani population. *Methods*. Patients with CHD (*n* = 310) and healthy individuals (*n* = 310) were enrolled in this study. Genotyping was performed by polymerase chain reaction-restriction fragment length polymorphism (PCR-RFLP). *Results*. A significant difference was observed in the −863C>A polymorphism between patients with CHD and control subjects (*P* < 0.0001). CHD risk was positively associated with the variant allele −863A (*P* < 0.0001) in the study subjects. There was no significant link between the −1031T>C polymorphism and CHD risk in the study population. Haplotypes A-T and A-C of the TNF-alpha gene loci at −863 and −1031 showed higher frequency in the patient group compared with controls (*P* < 0.05). *Conclusion*. The TNF-**α**  −863C>A gene polymorphism was associated with the pathogenesis of CHD while the −1031T>C polymorphism did not show any link with the disease in a Pakistani population.

## 1. Introduction

Coronary heart disease (CHD) is a leading health problem around the world. Atherosclerosis is a severe clinical manifestation of the disease [1]. Inflammation is a hallmark of CHD and other outcomes of atherosclerosis [1–3]. Inflammatory cytokines appear to stimulate the expression of mediators involved in the development of atherosclerosis and thromboembolic complications [4, 5]. Release of proinflammatory cytokines including the gene encoding TNF-*α* is regulated by several genes, which are thought to be involved in the pathogenesis of CHD. TNF-*α* gene is located on the human chromosome 6p21.3 and is arranged within class III region between MHC class II (HLA-II) and MHC class I antigens (HLA-I) [6, 7]. TNF-*α* gene induction is regulated by the p38 mitogen-activated protein kinase of the signaling cascade pathway [8].

TNF-*α* is one of the primary proinflammatory cytokines, mainly produced and secreted by inflammatory cells (i.e., monocytes and macrophages) [9, 10]. Evidence shows that TNF-*α* is a key contributor in the development, progression, and complications of atherosclerosis [11]. TNF-*α* is involved in reduced expression of endothelial nitric oxide synthase (eNOS) and thus impaired nitric oxide (NO) production leading to endothelial dysfunction [11, 12]. It has a profound effect on lipid metabolism and has been implicated in insulin resistance which produces changes in lipid and glucose associated with the cardiovascular disease risk [12, 13].

Genetic variants in the TNF-*α* promoter region are reported to be associated with the TNF-*α* serum levels [14–17]. These promoter polymorphisms regulate the transcriptional activity of TNF-*α* gene [18, 19]. Based upon these observations, the present study was designed to investigate the association of the −1031T>C (rs1799964) and −863C>A (rs1800630) polymorphisms of the promoter region of TNF-*α* gene with CHD in a Pakistani population. Prior to this study the said polymorphisms have not been investigated in the study population.

## 2. Methods

### 2.1. Study Population

This study was reviewed and approved by the Institutional Review Board (IRB), Quaid-i-Azam University, Islamabad. Informed consent was obtained from all participants of the study according to the Helsinki Declaration of 1975 as revised in 1997. A questionnaire was designed to characterize the study subjects.

We investigated 310 unrelated CHD patients (219 males and 91 females, mean age: 54.3 ± 10.15 years) attending hospitals in different regions of the country. The study participants were evaluated by clinical history, physical examination, 12-lead electrocardiography (ECG), and coronary angiography. ECG and coronary angiogram were analyzed by two cardiologists, who were unaware of the proposed study. Angiographic evidence showing >50% stenosis of at least one segment of a major coronary artery was defined as CHD [20]. 310 unrelated control subjects (222 males and 88 females, mean age: 53.2 ± 10.46 years) from the same geographic location were included in this study. The control subjects were clinically healthy with normal ECG and angiography. According to the revised criteria for Asian population, the category of overweight was defined as body mass index (BMI) of ≥23 Kg/m^2^ [21].

### 2.2. Sample Collection

Peripheral venous blood samples were obtained from the study population. For biochemical analysis, the blood samples were transferred into sterile plain serum tubes and the blood was allowed to clot. Then the samples were centrifuged to separate serum and the serum aliquots were stored at −80°C. For molecular analysis, blood samples were immediately transferred into ethylenediaminetetraacetic acid (EDTA) vacutainer tubes to prevent coagulation. Whole blood samples were used to extract genomic DNA using standard phenol-chloroform extraction method.

### 2.3. Biochemical Analyses

Biochemical analyses were carried out using AMP Diagnostics kits (Austria) for the determination of serum total cholesterol (TC), triglycerides (TG), low-density lipoprotein cholesterol (LDL-C), and high-density lipoprotein-cholesterol (HDL-C) on an automated analyzer (Vitalab Selectra E Chemistry Analyzer, The Netherlands).

### 2.4. DNA Extraction and Genotyping of the TNF-*α* Gene Promoter Polymorphism

The TNF-*α* gene −1031T>C polymorphism was investigated by the polymerase chain reaction (PCR) using forward primer 5′-TAT GTG ATG GAC TCA CCA GGT-3′ and reverse primer 5′-CCT CTA CAT GGC CCT GTC TT-3′. For the −863C>A polymorphism of the cytokine gene, forward primer 5′-GGC TCT GAG GAA TGG GTT AC-3′ and reverse primer 5′-CTA CAT GGC CCT GTC TTC GTT ACG-3′ were used for amplification. PCR amplification was carried out in 0.2 mL tubes (Axygen, CA, USA) in a total volume of 50 *μ*L. The reaction mixture contained 3 *μ*L of genomic DNA, 2.5 *μ*L of each forward and reverse primer (20 *μ*M stock), 5 *μ*L of 10X PCR buffer (200 mM of (NH_4_)_2_SO_4_, 750 mM of Tris-HCl (pH 8.8), and 0.1% Tween 20), 4 *μ*L of 25 mM MgCl_2_ (MBI-Fermentas, England), 1 *μ*L of 10 mM dNTPs (MBI-Fermentas, England), and 0.5 *μ*L (5 U/*μ*L) of *Taq* DNA polymerase (MBI-Fermantas, England) in 31.5 *μ*L of PCR water. PCR reactions were performed by means of GeneAmp PCR System 9700 (Applied Biosystems Inc, Foster City, CA, USA). PCR was carried out with the following thermal cycling conditions: an initial denaturation step at 94°C for 12 min, followed by amplification for 35 cycles at 94°C for 30 s, 59°C for 1 min, and 72°C for 2 min, followed by a final extension step at 72°C for 2 min. The PCR products were analyzed by 2% agarose gel electrophoresis.

*Bbs*I (MBI-Fermantas, England) restriction endonuclease was used for the detection of the TNF-*α* gene −1031T>C polymorphism. RFLP was performed in 0.2 mL tubes (Axygen, CA, USA) in a total volume of 20 *μ*L containing 12 *μ*L of amplified products, 2 *μ*L of 10X G buffer (10 mM Tris-HCl (pH 7.5), 10 mM MgCl_2_, 50 mM NaCl, and 0.1 mg/mL BSA), 0.3 *μ*L of *Bbs*I enzyme, and 5.7 *μ*L of PCR water. Digested products were analyzed by 3% agarose gel electrophoresis and visualized under ultraviolet light. Similarly, *Tai*I (MBI-Fermantas, England) enzyme was used to investigate the TNF-*α* gene −863C>A polymorphism using 10X R buffer (10 mM Tris-HCl (pH 8.5), 10 mM MgCl_2_, 100 mM KCl, and 0.1 mg/mL BSA). The digested products were subjected to 4% agarose gel electrophoresis and visualized under ultraviolet light.

### 2.5. Statistical Analysis

Statistical analysis was carried out using GraphPad Prism for Windows Version 5.00 (GraphPad Software, Inc., CA, USA). Basic and clinical variables were expressed as numbers and mean ± SD. Continuous variables were evaluated by Student's *t*-test. Genotype and allele frequencies were compared by Chi-square analysis. *P* value <0.05 was considered statistically significant.

## 3. Results

### 3.1. General Characteristics of the Study Population

The baseline and clinical characteristics of the study participants are shown in Table 1. Patients with CHD had significantly higher BMI (*P* = 0.002), systolic blood pressure (BP) (*P* < 0.0001), and lower HDL-C (*P* < 0.0001) than the control group. In this study, we did not find any significant difference in the levels of TC, TG, and LDL-C from patients with CHD and control subjects.

### 3.2. Genotype Analysis of the Study Population

The genotype frequencies of the TNF-*α* gene −1031T>C and −863C>A polymorphisms are shown in Table 2. The frequency of the TNF-*α*  −1031 variant genotype (TC + CC) was 41.29% in control individuals and 42.58% in CHD patients (OR = 1.05; 95% CI = 0.76–1.45; *P* = 0.80). There was no significant difference between the T and C alleles at −1031 from patients with CHD and controls (OR = 1.11; 95% CI = 0.85–1.43; *P* = 0.46).

At position −863 of TNF-*α* gene, the variant genotype (CA + AA) was more prevalent in the patient group (70.32%) compared with controls (35.16%) (OR = 4.37; 95% CI = 3.11–6.12; *P* < 0.0001). The frequency of the TNF-*α*  −863A allele was significantly higher in patients with CHD (35.65%) than in control subjects (18.87%) (OR = 2.38; 95% CI = 1.83–3.08; *P* < 0.0001).

Haplotypes A-T and A-C of the TNF-*α* gene loci at −863C>A and −1031T>C showed higher frequency in the patient group compared with controls (*P* < 0.05; Table 3).

## 4. Discussion

Several genetic components are involved in the pathophysiology of CHD. Studies on the involvement of TNF-*α* gene's promoter polymorphisms in its regulation are of legitimate clinical importance because of the potentially damaging proinflammatory effects of the cytokine. Relatively fewer studies have been conducted on the TNF-*α* gene  −1031T>C and −863C>A polymorphisms in relation to CHD. Therefore, in the present case-control study, we have investigated the association of single nucleotide polymorphisms (SNPs and TNF-*α*  −1031T>C and −863C>A) with CHD in a Pakistani population.

Our findings suggest that the TNF-*α* gene −1031T>C polymorphism had no measurable influence on the occurrence of CHD in the study population. The TNF-*α*–1031C allele frequency was 23.55% in control individuals versus 25.48% in patients with CHD (*P* = 0.46), indicating that the −1031C allele is not a risk factor of CHD in our population. This is in accordance with the results of Ghazouani et al. who have demonstrated no link between the TNF-*α*  −1031T>C promoter polymorphism and coronary artery disease (CAD) in a Tunisian population [22]. Oda et al. have reported that the TNF-*α*  −1031C allele acts as a protective factor in atherosclerotic severity [23]. Our results are also in agreement with the findings of Bennet and associates in Swedish patients with myocardial infarction [15] and Liang et al. in Chinese patients with DCM [24]. Interestingly, Kamoun and colleagues have reported a link between the TNF-*α*  −1031T>C polymorphisms with another inflammatory condition in a Tunisian population [25]. Genetic heterogeneity exists among different ethnic groups around the world, and, therefore, it is likely that the −1031T>C SNP may be associated with certain clinical phenotype in some ethnic groups.

We have observed a significant difference in the TNF-*α* gene −863C>A polymorphism between the patients and control group (*P* < 0.0001). The −863A allele was more common in patients (35.65%) as compared to 18.87% in healthy controls (*P* < 0.0001). Higher frequency of the variant allele (−863A) indicates a significant association of this polymorphism with CHD in the studied subjects. A study carried out by Xiang and colleagues in a Chinese population also revealed a significant difference in the cytokine's genotype and allele frequencies at −863 between patients and controls [26]. In contrast to these findings, some studies reported a nonsignificant difference in the −863C/A genotype frequency between patients with CHD, myocardial infarction, and cardiomyopathy versus healthy controls [15, 24, 27]. Furthermore, no association was observed between the −863C>A polymorphism and cardiovascular mortality in the German population [28]. Although a number of studies have documented a lack of link between the TNF-*α*  −863C>A polymorphism and cardiovascular disease, in the current study we have observed a strong association of the said polymorphism with CHD in a Pakistani population. South Asians are considered to be at an increased risk of cardiovascular events. It is probable that genetic factors may affect the disease phenotype differently among different ethnic groups. The current study suggests that the −863A allele carriers in the study population could be at an increased risk of cardiovascular complications.

Polymorphism in the promoter region of TNF-*α* gene may be involved in the transcriptional regulation by means of modification in the transcription factor binding sites [29]. The −1031C and −863A variants have been found to be associated with the increased TNF-*α* gene expression [30]. Factors binding to the regulatory elements, such as nuclear factor *κ*B (NF- *κ*B) and organic cation transporter-1 (OCT-1), and variations in the secondary structure of DNA motifs are the factors that influence the accession of *cis*-acting transcription factors to the promoter region of TNF-*α* gene [14, 31]. Evidence shows that during lipopolysaccharide stimulation, the TNF-*α*  −863A allele confers increased promoter activity as compared to the −863C allele [32]. Skoog et al. observed that the TNF-*α*  −863C>A gene polymorphism is involved in the allele specific chromatin remodeling of the cytokine's gene promoter region and recruitment of transcription factors which resulted in the increased promoter activity [33]. These lines of evidence suggest that a nucleotide variation from C>A at −863 of TNF-*α* gene may result in enhanced levels of the inflammatory cytokine in circulation that could affect the pathophysiology of CHD.

Based on haplotype analysis (Table 3), we have observed that A-T and A-C of the TNF-*α* −863C>A and −1031T>C gene loci were significantly associated with CHD in the study population. It seems probable to assume that the variant alleles at the investigated loci of TNF-*α* gene may confer functional changes in patients with CHD which may result in the increased levels of the cytokine in circulation that could have implications in the disease pathophysiology. However, these findings should be treated cautiously as more in-depth studies would be required to replicate the observations from the current study in other ethnic groups.

## 5. Conclusion

A significant association was observed between the TNF-*α* gene −863C>A promoter polymorphism and CHD; the cytokine's SNP may have a role in the pathogenesis of the disease. In this study the TNF-*α* gene  −1031T>C polymorphism was not linked with CHD.

## Figures and Tables

**Table 1:** Baseline and clinical characteristics of the study population.

Characteristics	Controls (*n* = 310)	Patients (*n* = 310)	*P* value^a^
Age (years)	53.2 ± 10.46	54.3 ± 10.15	0.164
BMI (Kg/m^2^)	25.1 ± 3.21	25.9 ± 3.43	0.002*
Systolic BP (mmHg)	120.6 ± 10.31	132.9 ± 26.20	<0.0001*
Diastolic BP (mmHg)	83.2 ± 6.28	84.7 ± 13.43	0.07
Cholesterol (TC) (mg/dL)	167.57 ± 29.08	161.48 ± 51.85	0.072
Triglycerides (TG) (mg/dL)	150.23 ± 57.02	143.29 ± 72.80	0.187
LDL-C (mg/dL)	97.41 ± 33.21	95.96 ± 36.11	0.603
HDL-C (mg/dL)	40.07 ± 9.68	35.73 ± 13.38	<0.0001*

Values are given as means ± SD.

^a^ *P* value calculated by Student's *t*-test (unpaired).

*P value (<0.05) indicates statistical significance.

*n*: number; SD: standard deviation.

**Table 2:** Genotype and allele frequencies of the TNF-*α* gene −1031T>C and −863C>A polymorphisms in the study population.

TNF-α SNP	Controls (*n* = 310)	Patients (*n* = 310)	OR^†^	95% CI^‡^	*P* value^a^
−1031T/C					
TT	182 (58.71%)	178 (57.42%)	1.05	0.76–1.45	0.8
TC + CC	128 (41.29%)	132 (42.58%)			
T	474 (76.45%)	462 (74.52%)	1.11	0.85–1.43	0.46
C	146 (23.55%)	158 (25.48%)			
−863C/A					
CC	201 (64.84%)	92 (29.68%)	4.37	3.11–6.12	<0.0001*
CA + AA	109 (35.16%)	218 (70.32%)			
C	503 (81.13%)	399 (64.35%)	2.38	1.83–3.08	<0.0001*
A	117 (18.87%)	221 (35.65%)			

Values are given in numbers and percentage.

^a^ *P* value calculated by Chi-square test.

*P value (<0.05) indicates statistical significance.

^†^Odds ratio.

^‡^95% confidence intervals.

**Table 3:** Haplotype analysis of TNF-*α* −1031T>C and −863C>A polymorphisms in the study population.

TNF-*α* −863C/A, TNF-*α* −1031T/C	Controls *n* (% age) 310 (100)	Patients *n* (% age) 310 (100)	*χ* ^2^	95% CI	*P* value
C-T	433 (70)	355 (57)	Reference		
A-T	39 (6)	110 (18)	40.36	3.44(2.326–5.088)	<0.0001*
C-C	69 (11)	44 (7)	1.259	0.77(0.519–1.164)	0.226
A-C	79 (13)	111 (18)	10.44	1.71(1.243–2.362)	0.001*

*P* value calculated by Chi-square stest (Yates corrected).

*P value (<0.05) indicates statistical significance.

*n*: number; OR: odds ratio; CI: confidence interval.
